# Association of *PCSK1* rs6234 with Obesity and Related Traits in a Chinese Han Population

**DOI:** 10.1371/journal.pone.0010590

**Published:** 2010-05-17

**Authors:** Qibin Qi, Huaixing Li, Ruth J. F. Loos, Chen Liu, Frank B. Hu, Hongyu Wu, Zhijie Yu, Xu Lin

**Affiliations:** 1 Key Laboratory of Nutrition and Metabolism, Institute for Nutritional Sciences, Shanghai Institutes for Biological Sciences, Chinese Academy of Sciences and Graduate School of the Chinese Academy of Sciences, Shanghai, China; 2 MRC Epidemiology Unit, Institute of Metabolic Science, Addenbrooke's Hospital, Cambridge, United Kingdom; 3 Department of Nutrition, Harvard School of Public Health, Boston, Massachusetts, United States of America; National Institute of Child Health and Human Development/National Institutes of Health, United States of America

## Abstract

**Background:**

Common variants in *PCSK1* have been reported to be associated with obesity in populations of European origin. We aimed to replicate this association in Chinese.

**Methodology/Principal Findings:**

Two *PCSK1* variants rs6234 and rs6235 (in strong LD with each other, r^2^ = 0.98) were genotyped in a population-based cohort of 3,210 Chinese Hans. The rs6234 was used for further association analyses with obesity and related traits. We found no significant association of rs6234 with obesity, overweight, BMI, waist circumference, or body fat percentage (*P*>0.05) in all participants. However, the rs6234 G-allele showed a significant association with increased risk of combined phenotype of obesity and overweight (OR 1.21[1.03–1.43], *P* = 0.0193) and a trend toward association with obesity (OR 1.25[0.98–1.61], *P* = 0.08) in men, but not in women (*P*≥0.29). Consistently, the rs6234 G-allele showed significant association with increased BMI (*P* = 0.0043), waist circumference (*P* = 0.008) and body fat percentage (*P* = 0.0131) only in men, not in women (*P*≥0.24). Interestingly, the rs6234 G-allele was significantly associated with increased HOMA-B (*P* = 0.0059) and decreased HOMA-S (*P* = 0.0349) in all participants.

**Conclusion/Significance:**

In this study, we found modest evidence for association of the *PCSK1* rs6234 with BMI and overweight in men only but not in women, which suggested that *PCSK1* rs6234 might not be an important contributor to obesity in Chinese Hans. However, further studies with larger sample sizes are needed to draw a firm conclusion.

## Introduction

The *PCSK1* (prohormone convertase 1/3) gene encodes prohormone convertase 1/3 that converts inactive prohormones (including proinsulin, proglucagon and proopiomelanocortin) into biologically active peptide hormones. Loss-of-function mutations in *PCSK1* gene cause monogenic obesity and impaired glucose tolerance in human [1], [2], [3], implying that common variants in *PCSK1* might predispose to obesity and type 2 diabetes in the general population. Recently, the *PCSK1* nonsynonmous variants rs6232 (encoding N221D) and rs6234-rs6235 (encoding the Q665E-S690T pair) have been shown to be consistently associated with obesity risk (OR = 1.34 and 1.22, respectively) in European populations including a total of 13,659 individuals from eight independent case-control or family-based cohorts [4]. However, only a modest evidence for association between body mass index (BMI) and the *PCSK1* variant rs6232 (*P* = 0.03) was found in a meta-analysis of 15 genome-wide association studies (GWAS) for BMI with 32,387 individuals of European origin [5]. A subsequent study in 3,885 non-diabetic Swedes failed to confirm the association between the *PCSK1* rs6235 and obesity [6]. Similarly, another recent study in 20,249 individuals of European descents from Norfolk, UK, also failed to replicate the association of the *PCSK1* rs6235 with obesity and related traits, and only found a suggestive evidence for association of the *PCSK1* rs6232 with obesity and BMI in younger individuals (age <59 yrs) but not in older age group [7]. Due to the inconsistent results, further replication studies are essential to confirm the association between genetic variation in *PCSK1* and obesity, particularly in other ethnic populations. In this study, we aimed to examine whether the previously reported association of the *PCSK1* genetic variants with obesity and related traits could be replicated in a population-based cohort of Chinese Hans including 3,210 unrelated individuals from Beijing and Shanghai.

## Materials and Methods

### Study population

The study sample consisted of 3,210 individuals (1,423 men and 1,787 women) from the Study on Nutrition and Health of Aging Population in China, a population-based study among non-institutionalized and unrelated Chinese Hans aged 50 to 70 years from Beijing and Shanghai. The study population, design and protocols have been described in detail elsewhere [8]. Height, weight, waist circumference and body fat percentage, as well as fasting glucose and insulin were measured following standard protocols as described previously [8]. Homeostasis model assessment of pancreatic beta-cell function (HOMA-B) and insulin sensitivity (HOMA-S), as measures of beta-cell function and insulin sensitivity, respectively, were estimated by the homeostasis model using Levy's computer model [9]. Normal weight, overweight and obesity were defined as BMI <24, 24≤ BMI ≤28 and BMI ≥28 kg/m^2^, respectively, according to the Chinese criteria [10]. The study was approved by the Institutional Review Board of the Institute for Nutritional Sciences, Shanghai, China, with written informed consent from all participants. The phenotypic characteristics of the population are shown in Table 1.

**Table 1:** Characteristics of the study population.

	Men	Women	All
N (%)	1,423 (44.3)	1,787 (55.7)	3,210
Age (year)	58.8±5.9	58.4±6.1	58.6±6.0
BMI (kg/m^2^)	24.0 (21.8–26.3)	24.5 (22.1–26.9)	24.2 (22.0–26.6)
Waist circumference (cm)	85.6 (78.0–92.7)	81.7 (74.9–89.0)	83.5 (76.0–90.8)
Body fat percentage (%)^a^	20.4±5.4	32.5±5.3	27.5±8.0
Fasting glucose (mmol/L)	5.95±1.89	5.74±1.59	5.8±1.74
Fasting insulin (pmol/L)	74.4 (52.8–102.0)	88.2 (64.8–119.4)	82.2 (59.4–112.2)
HOMA-B (%)	101.6±45.6	117.2±46.9	110.3±47.0
HOMA-S (%)	69.5 (51.1–96.6)	59.3 (44.6–81.1)	63.6 (47.1–86.9)
Overweight (%)	531 (37.3)	693 (38.7)	1224 (38.1)
Obese (%)	177 (12.4)	297 (16.6)	474 (14.8)
IFG (%)	425 (29.9)	453 (25.4)	878 (27.4)
Type 2 diabetes (%)	207 (14.6)	217 (12.1)	424 (13.2)

Data are n (%), means±SD or medians (interquartile range).

BMI, body mass index; HOMA-B, homeostasis model assessment of pancreatic beta-cell function; HOMA-S, homeostasis model assessment of insulin sensitivity; IFG, impaired fasting glucose.

a Body fat percentage (%) was assessed using the dual-energy X-ray absorptiometry (DEXA) among 1,634 participants (711 men and 923 women) from Shanghai.

### Genotyping

Genomic DNA was extracted from peripheral blood leukocytes by salting out procedure (http://www.protocol-online.org/prot/Detailed/3171.html). Single nucleotide polymorphism (SNP) genotyping was performed with TaqMan SNP allelic discrimination by the ABI PRISM 7900HT Sequence Detection System (Applied Biosystems) according to the manufacturer's protocol. Since the *PCSK1* rs6232 is largely monomorphic in Chinese (minor allele frequency [MAF] = 0.01 and 0 for HapMap-CHB sample and AFD_CHN_PANEL, respectively), only the rs6234 and rs6235 were successfully genotyped in this study. The genotyping success rates of the rs6234 and rs6235 were 98.5% and 98.3%, respectively, and the concordance rates were both >99.5% based on 12% duplicate samples (n = 384). The allele frequencies of both SNPs (MAF = 0.330 and 0.328 for rs6234 and rs6235 in current sample, respectively) were comparable to those in the HapMap HCB database, and in Hardy-Weinberg equilibrium (*P*>0.54). Since these 2 SNPs were in almost complete linkage disequilibrium (LD) (r^2^ = 0.98 in this population and r^2^ = 1 in HapMap-CHB population), only the rs6234 was selected for further association analyses as it had a higher genotyping success rate.

### Statistical analysis

The association between the rs6234 and risk of overweight, obesity and type 2 diabetes was tested by logistic regression under an additive model, adjusting for age, gender, geographical region (Shanghai/Beijing) and BMI (where appropriate). Generalized linear regression was applied to test for association between the rs6234 and quantitative traits in which participants with known diabetes or receiving glucose-lowering treatment (n = 267) were excluded. BMI, waist circumference, insulin and HOMA-S were natural log-transformed before analyses. Since the SNP distribution was not different between geographical regions (*P*>0.88), we pooled all 3,210 individuals for the association studies. SNP-gender and SNP-age (<60 yrs, and >60 yrs) interactions were assessed by including the respective interaction term in the regression models. LD was estimated using Haploview V3.2 (http://www.broad.mit.edu/mpg/haploview). All reported *P*-values are nominal and two-sided. Association analyses were performed with SAS version 9.1 (SAS Institute, Cary, North Carolina). Power calculations were performed using the Quanto software (http://hydra.usc.edu/gxe/).

## Results

We found no significant association between *PCSK1* rs6234 risk G-allele and obesity or overweight among all participants (*P*≥0.10) (Table 2). However, the *PCSK1* rs6234 G-allele showed a significant association with increased risk of combined phenotype of obesity and overweight (OR 1.21[1.03–1.43], *P* = 0.0193) (*P* for SNP-gender interaction  = 0.11) and a trend toward association with increased obesity risk (OR 1.25[0.98–1.61], *P* = 0.08) (*P* for SNP-gender interaction  = 0.0458) in men, but not in women (*P*≥0.29) in the gender-stratified analyses (Table 2). Consistently, the rs6234 G-allele was significantly associated with increased BMI (*P* = 0.0043) (*P* for SNP-gender interaction  = 0.0045), waist circumference (*P* = 0.008) (*P* for SNP-gender interaction  = 0.0055) and body fat percentage (*P* = 0.0131) (*P* for SNP-gender interaction  = 0.08) only in men, whereas no associations were observed in women (*P*≥0.24). Our study had only 72.5% power to detect OR = 1.22 for obesity, as previously reported by the original study [4], at a significance of 5% and a MAF ≥34%. We, therefore, cannot rule out the possibility of false negative findings in women. In addition, no SNP-age interactions on risk of obesity or overweight or related traits were observed.

**Table 2:** Case-control analyses of *PCSK1* rs6234 with obesity and overweight.

	Major/minor allele	Obesity vs. Normal				Obesity and overweight vs. Normal			
		n/MAF		OR (95% CI)	*P*	n/MAF		OR (95% CI)	*P*
		Cases	Controls			Cases	Controls		
All	C/G	471/0.321	1489/0.319	1.02 (0.86–1.19)	0.86	1674/0.339	1489/0.319	1.10 (0.98–1.22)	0.10
Men		176/0.366	703/0.312	1.25 (0.98–1.61)	0.08	696/0.360	703/0.312	1.21 (1.03–1.43)	0.0196
Women		295/0.293	786/0.324	0.89 (0.72–1.10)	0.29	978/0.324	786/0.324	1.02 (0.88–1.18)	0.82
*P* for interaction					0.0458				0.11

The ORs are odds ratios that represent the effects of risk allele (G-allele) based on an additive model, in which individuals homozygous for CC were coded as 0, heterozygous individuals CG were coded as 1, and individuals homozygous for GG were coded as 2; The ORs and *P* values were adjusted for age, region and sex (where appropriate).

MAF, minor allele frequency.

We observed no association with the risk of type 2 diabetes or combined type 2 diabetes/IFG either in all individuals or in men and women separately (*P*>0.50) (data not shown). In quantitative traits analyses, the rs6234 G-allele was significantly associated with increased HOMA-B (*P* = 0.0059) and decreased HOMA-S (*P* = 0.0349), but not with fasting glucose (*P* = 0.93). No SNP-gender interactions (*P*≥0.16) were observed (Table 3).

**Table 3:** Quantitative traits analyses of *PCSK1* rs6234 with obesity related traits.

	*PCSK1* rs6234				
	CC	CG	GG	*P*	*P* for interaction
BMI (kg/m^2^)^a^ ^b^					
All	24.0 (0.1)	24.1 (0.1)	24.2 (0.2)	0.45	0.0045
Men	23.5 (0.1)	23.9 (0.1)	24.3 (0.3)	0.0043	
Women	24.5 (0.1)	24.4 (0.1)	24.1 (0.3)	0.26	
Waist circumference (cm)^a^ ^b^					
All	82.8 (0.3)	83.1 (0.3)	83.2 (0.6)	0.49	0.0055
Men	83.8 (0.4)	85.9 (0.4)	86.8 (0.8)	0.008	
Women	81.5 (0.4)	81.3 (0.4)	80.6 (0.8)	0.24	
Body fat percentage (%)^b^					
All	26.0±0.2	26.7±0.3	26.7±0.5	0.06	0.08
Men	19.7±0.4	20.8±0.4	21.5±0.8	0.0131	
Women	32.3±0.3	32.7±0.3	32.2±0.6	0.72	
Glucose (mmol/L)^c^					
All	5.57±0.03	5.62±0.03	5.53±0.07	0.93	0.79
Men	5.62±0.05	5.68±0.05	5.59±0.11	0.83	
Women	5.52±0.04	5.56±0.04	5.47±0.09	0.93	
Insulin (pmol/L)^a^ ^c^					
All	77.9 (1.1)	81.8 (1.1)	79.0 (2.2)	0.09	0.31
Men	71.5 (1.5)	73.7 (1.5)	70.4 (3.1)	0.83	
Women	83.2 (1.4)	89.1 (1.6)	86.1 (3.1)	0.0441	
HOMA-B (%)^c^					
All	111.1±1.2	114.4±1.2	117.6±2.5	0.0059	0.16
Men	105.5±1.8	105.5±1.8	109.9±3.7	0.43	
Women	116.9±1.6	122.8±1.6	125.2±3.3	0.0032	
HOMA-S (%)^a^ ^c^					
All	67.6 (0.9)	64.4 (0.8)	65.6 (1.8)	0.0349	0.61
Men	72.5 (1.5)	71.0 (1.4)	70.0 (2.9)	0.37	
Women	63.9 (1.1)	59.4 (1.0)	62.4 (2.2)	0.0492	

Data are means±SE or geometric means (SE) unless otherwise indicated; Participants previously diagnosed type 2 diabetes or receiving anti-glucose treatment (n = 267) were excluded from the analyses.

BMI, body mass index; HOMA-B, homeostasis model assessment of pancreatic beta-cell function; HOMA-S, homeostasis model assessment of insulin sensitivity.

a These variables were log-transformed before analyses.

b Adjusted for age, region and sex (where appropriate).

c Adjusted for age, region, BMI and sex (where appropriate).

## Discussion

In this population-based sample of Chinese Hans, we did not replicate the association between the *PCSK1* rs6234-rs6235 pair and obesity risk previously reported by original study in European populations [4]. This is consistent with findings of several recent studies including 2 independent GWAS in White Europeans, in which no association was found between the *PCSK1* rs6235 and obesity or BMI [5], [6], [7], [11]. Interestingly, we found that the *PCSK1* rs6234 risk G-allele was marginally associated with obesity and significantly associated with combined obesity/overweight in men, but not in women when the analyses were stratified by gender. Consistently, the same risk allele was also significantly associated with increased BMI and waist circumference in men in the quantitative trait analyses, but not in women, with significant SNP-gender interactions on BMI (*P* for interaction  = 0.0045) and waist circumference (*P* for interaction  = 0.0055). This is the first study to report the significant SNP-gender interactions of the *PCSK1* rs6234 on obesity related traits in Chinese Hans. We therefore choose to report all the results for men and women separately to highlight all potentially useful associations for subsequent studies. However, two previous replication studies in populations of European origin did not find any SNP-gender interaction of the *PCSK1* rs6235 on obesity risk [6], [7]. Moreover, a more recent study in 1,094 subjects of Chinese origin has suggested that a common variant (rs155791) in strong LD with the rs6235 and rs6234, and two common haplotypes were all associated with obesity [12]. Lack of replication of the previously reported association in women could be because of limited power of this current study. Nevertheless, further studies with larger sample sizes would be required to provide definite evidence for the association between common variants in *PCSK1* and obesity in Chinese Hans.

Another interesting finding of the present study is that the *PCSK1* rs6234 G-allele was significantly associated with decreased insulin sensitivity and increased beta-cell function estimated by HOMA-S and HOMA-B, respectively. The underlying mechanism remained to be investigated. Consistently, the recent study in a Chinese population has also found a suggestive evidence for association between the *PCSK1* rs6235 and increased HOMA-IR values (*P* = 0.02) [12]. The compensatory adaptation of pancreatic beta-cells to insulin resistance might lead to increased HOMA-B. However, more studies are warranted to confirm this hypothesis.

In conclusion, in this population-based cohort of Chinese Hans, we found modest evidence for association of the *PCSK1* variant rs6234 with BMI and overweight in men only, but not in women. Further studies with larger sample sizes are warranted to confirm the association between common variants in *PCSK1* and obesity risk in Chinese Hans, especially in women.
